# Supplementary material for: Impact of interface traps on charge noise and low-density transport properties in Ge/SiGe heterostructures
Source: Commun Mater. 2024 Aug 14;5(1):151. doi: 10.1038/s43246-024-00563-8 (PMC11324522; doi:10.1038/s43246-024-00563-8)
Supplement: Supplementary file 2 — Supplementary Information [file 43246_2024_563_MOESM2_ESM.pdf]

Supplementary Information: Impact of interface traps on charge noise and low-density transport properties in Ge/SiGe heterostructures

Leonardo Massai,[1,*] Bence Hetényi,[1] Matthias Mergenthaler,[1] Felix J. Schupp,[1] Lisa Sommer,[1] Stephan Paredes,[1] Stephen W. Bedell,[2] Patrick Harvey-Collard,[1] Gian Salis,[1] Andreas Fuhrer,[1,†] and Nico W. Hendrickx[1]

[1] *IBM Research Europe - Zurich, Säumerstrasse 4, 8803 Rüschlikon, Switzerland*

[2] *IBM Quantum, T.J. Watson Research Center, 1101 Kitchawan Road, Yorktown Heights, New York 10598, USA*

(Dated: June 25, 2024)

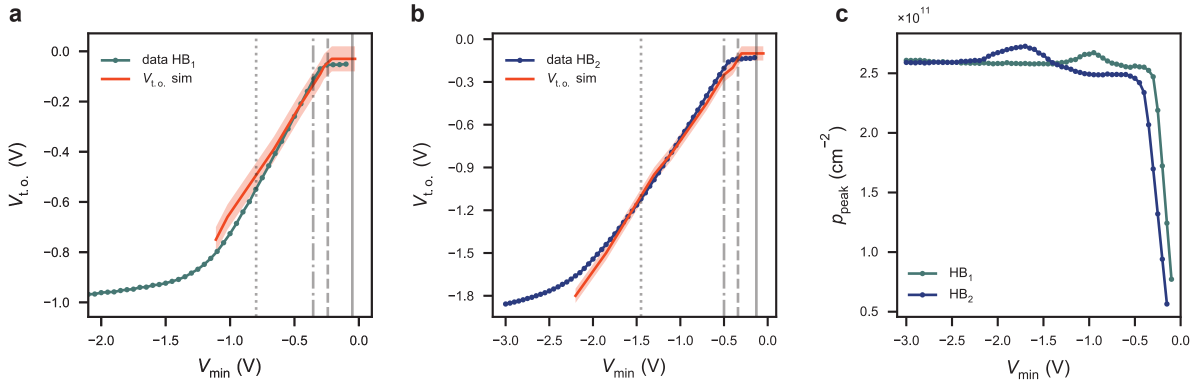

Supplementary Figure 1. **Simulation of the channel turn-on curves**: **a**, Simulated (red) and measured (green) turn-on voltage of HB_1 as a function of minimum gate voltage. **b**, Simulated (red) and measured (blue) turn-on voltage of HB_2 as a function of minimum gate voltage. Error bars for the turn-on curves arise from the finite resolution of the applied gate voltage in the simulation. **c**, Peak density as a function of $V_{\rm min}$ measured in HB_1 and HB_2, reached when $V_{\rm g} = V_{\rm min}$. The density in the QW saturates near $p_{\rm QW,sat} \sim 2.5 \times 10^{11}$ cm^{-2}, similar for both HBs.

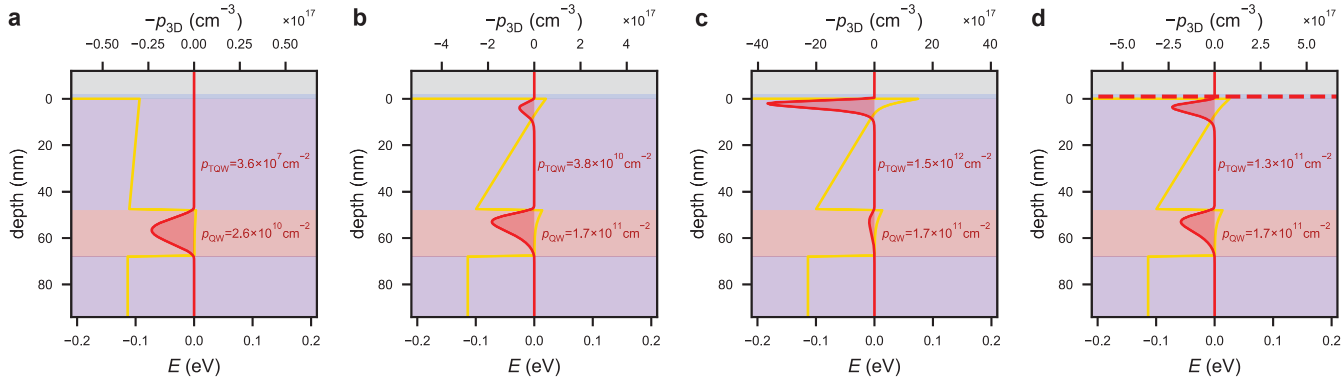

Supplementary Figure 2. **Simulation of the valence band edge and hole density in the heterostructure**: **a**, Holes are accumulating only in the Ge channel for $V_{\rm g} = -0.15$ V. **b**, Holes are starting to accumulate at the SiGe-SiO_2 interface due to Fowler-Nordheim tunnelling at $V_{\rm g} = -0.35$ V. **c**, Substantial hole density ($p_{\rm TQW} = 1.5 \times 10^{12}$ cm^{-2}) is accumulating at the SiGe-SiO_2 interface for $V_{\rm g} = -1.45$ V. **d**, Modified band structure at $V_{\rm g} = -1.45$ V assuming a trapped charge density of $p_{\rm i.t.} = 1.5 \times 10^{12}$ cm^{-2} at the position highlighted by the dashed red line.

* lem@zurich.ibm.com

† afu@zurich.ibm.com

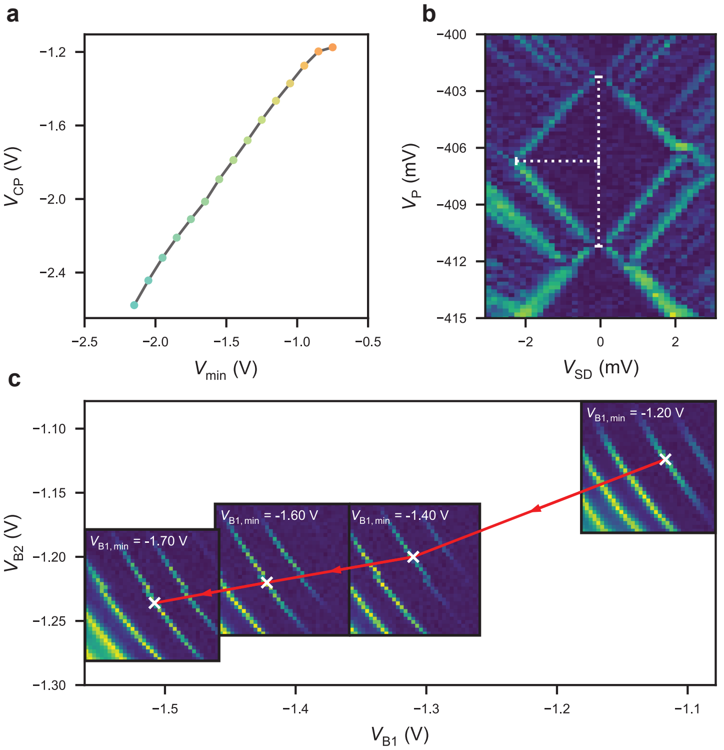

Supplementary Figure 3. **QD characterisation**: **a**, Effective Coulomb-peak voltage position V_{CP} as defined in the Methods as a function of the minimum gate voltage set on gate P. **b**, Measurement of the differential conductance $dI_{\mathrm{SD}}/dV_{\mathrm{SD}}$ for a Coulomb diamond in the few-hole regime. The extracted lever arm for plunger P is $\alpha_{\mathrm{P}} \approx 0.23$. **c**, Coulomb resonances in the few-hole regime used to extract the locality of charge traps in Section II D. As the voltage on B1 is cyclically pushed to $V_{\mathrm{B1,min}}$ (following the red arrows), the CPs position shifts moderately in V_{B2} and predominantly in V_{B1}. $V_{\mathrm{B1,CP}}$ and $V_{\mathrm{B2,CP}}$ reported in Fig. 5f are extracted at symmetric dot-lead tunnel rates (white crosses) for each $V_{\mathrm{B1,min}}$.

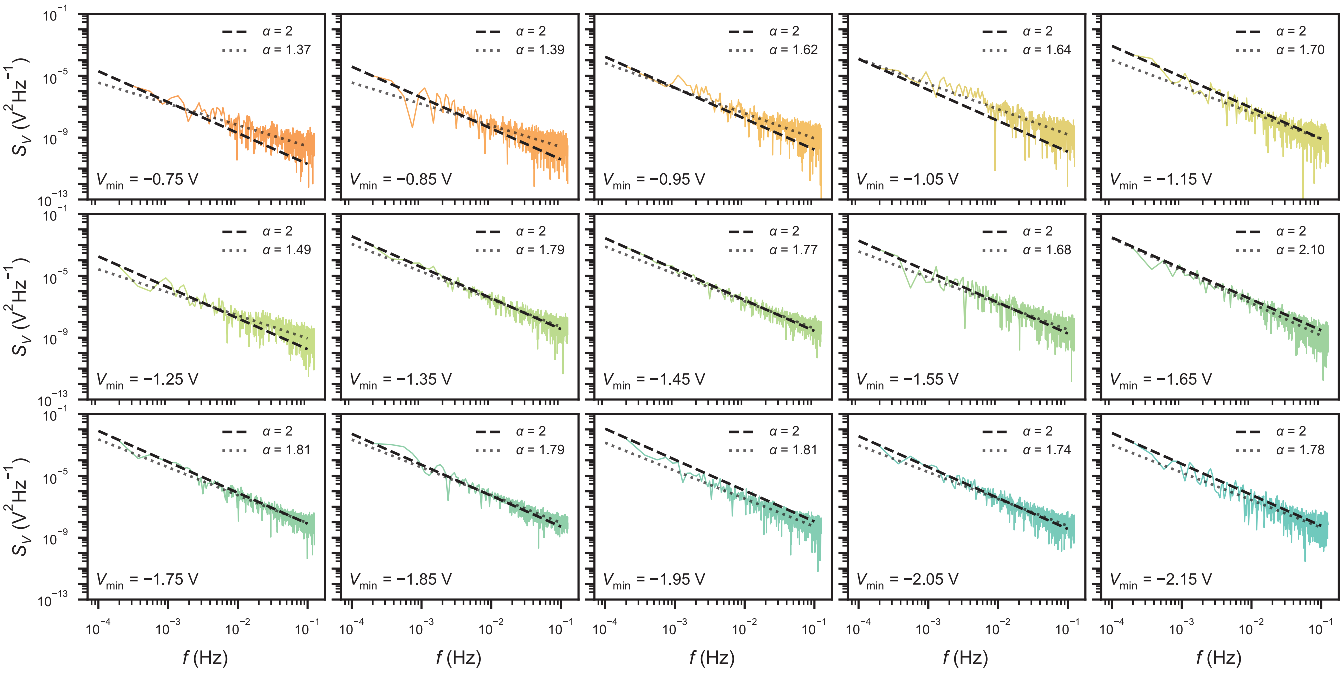

Supplementary Figure 4. **Charge noise measurement and data analysis in QD**: Power spectral density S_V of the Coulomb peak position $V_{\mathrm{P,CP}}(t)$ for the different V_{min} with $1/f^{\alpha}$ fits with fixed or free exponent α, respectively dashed and dotted lines.

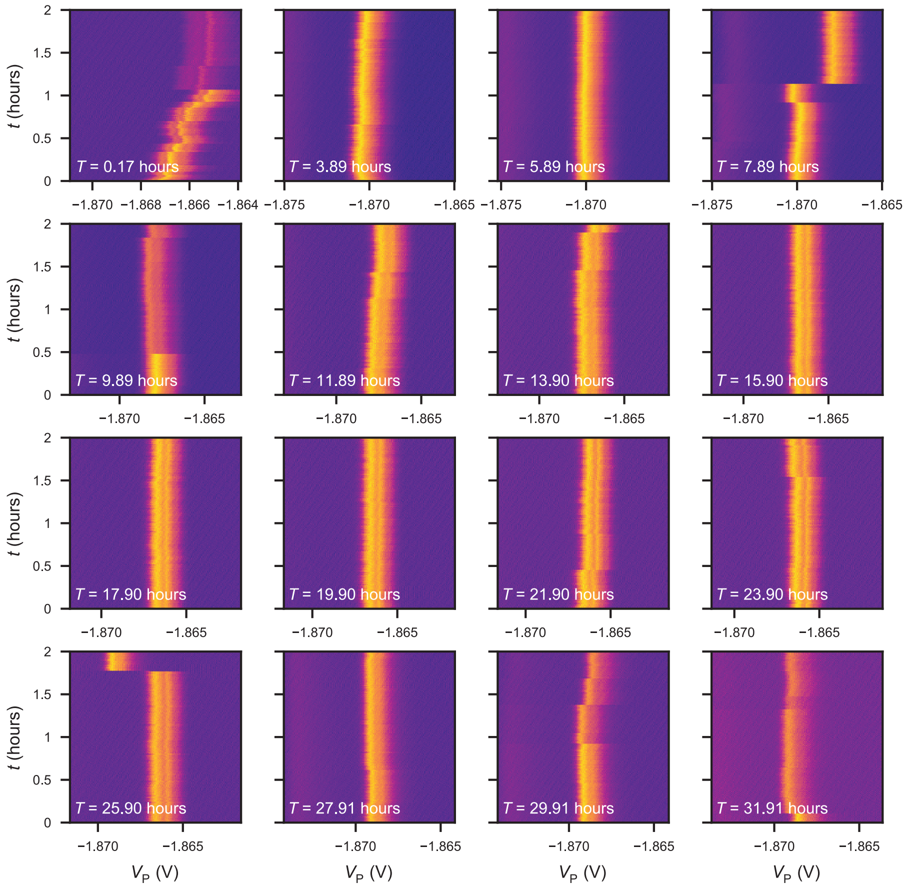

Supplementary Figure 5. **CPT measurements after setting V_P to $V_\mathrm{min} = -2.05$ V**: Each plot is a 2-hour-long CPT measurement starting at time T (white text) after pushing V_P to $V_\mathrm{min} = -2.05$ V. The data sets taken at $T = 7.89$ hours (top right plot) and $T = 25.90$ hours (bottom left plot) show substantial jumps in $V_\mathrm{P,CP}$, leading to a strongly increased noise spectral density at low frequencies, as observed in Fig. 5e.
